# Influenza A virus infection dynamics in two sow herds and effects of interventions

**DOI:** 10.1186/s40813-025-00481-2

**Published:** 2026-01-13

**Authors:** Marianne Viuf Agerlin, Lars Erik Larsen, Nicolai Rosager Weber, Mette Fertner, Nicole Bakkegård Goecke, Pia Ryt-Hansen

**Affiliations:** 1 Department of Veterinary Animal Sciences, University of Copenhagen, Dyrlaegevej 88, Frederiksberg, 1870 Denmark; 2 Department of Veterinary and Quality Services, Danish Agriculture and Food Council F.m.b.A, Axeltorv 3, Copenhagen, Denmark; 3 Department of Statistics & Analyses, SEGES Innovation P/S, Axeltorv 3, Copenhagen, Denmark

**Keywords:** Swine influenza A virus, SwIAV, Shedding, Piglets and weaned pigs, Haemagglutination inhibition, Maternal antibody, ELISA antibody titer, Interventions, Weight

## Abstract

**Background:**

The epidemiology of swine influenza A virus (swIAV) has undergone a change from causing mainly epidemic infections to continuous endemic circulation within herds. Several studies have traced the within-herd swIAV transmission pattern. However, studies with more frequent samplings of targeted age groups are needed to increase the current understanding of virus transmission dynamics. In addition, data on the efficacy of different intervention methods for reducing swIAV spread are highly relevant for disrupting endemic swIAV infections. Therefore, this study aimed to investigate the weekly influenza infection dynamics and evaluate subsequent interventions, including vaccination and management changes.

**Method:**

Two batches of pigs in two sow herds (Herd 1 and 2) were sampled with nasal swabs and blood samples from one week of age to the end of the nursery period. In each batch, ten sows were included with each six piglets including three cross-fostered and three of the sows own piglets. Nasal swabs were tested for the presence of swIAV, and blood samples were tested for swIAV antibodies using ELISA and HI-test. In Herd 2, interventions including pre-farrowing vaccination, limited cross-fostering, litter-wise weaning and disinfection of tools and hands were implemented during a second round of sampling in two batches as described above.

**Results:**

In both herds, an endemic presence of swIAV was observed, with Herd 1 having one circulating swIAV subtype (H1C.2.4N2G) circulating in the farrowing unit and Herd 2 having two swIAV strains (H1C.2.4N2G and H1N1pdm09) circulating both before and after weaning. Maternally derived swIAV antibodies were successfully transferred from sow to piglets, but despite high levels, swIAV infections was observed in the piglets down to one week of age and a lack of subsequent seroconversion was observed. The implementation of pre-farrowing vaccination increased the level of swIAV antibodies in sows and piglets but had no obvious effect on viral shedding and clinical disease.

**Conclusions:**

The endemic state of swIAV infections in sow herds was clearly illustrated in this study along with the difficulties in controlling swIAV through vaccinations and management changes in an environment that favours virus circulation.

**Supplementary Information:**

The online version contains supplementary material available at 10.1186/s40813-025-00481-2.

## Introduction

Influenza A virus in swine (swIAV) has become endemic in swine herds [1–6]. Since the 2009 pandemic, an increasing number of human IAV introductions into swine herds have been reported and several novel swIAV strains and genotypes have emerged along with a higher incidence of variant cases in humans [7–9]. SwIAV causes respiratory disease in pigs, with nasal discharge, conjunctivitis, coughing, sneezing, fever and lethargy as the dominant clinical signs. Infected pigs have a reduced weight gain and feed conversion rate, extending the time from birth to slaughter [4, 10–14]. In addition, swIAV predisposes for secondary infections, and is one of the most important pathogens in the porcine respiratory disease complex (PRDC) [15–19] posing significant economic and animal health concerns globally [4, 20, 21].

At present, there is a need for a better understanding of which risk factors are most important for swIAV circulation and persistence within herds [2, 18, 19]. These risk factors are related to a range of factors affecting the pigs, including the level of external and internal biosecurity, housing conditions and circulation of other pathogens. Furthermore, management factors such as the use of cross-fostering and nurse sows can also contribute to the intra-herd spread of the virus [22]. In individual pigs, the level of pre-existing immunity likely plays an important role in determining susceptibility to infection. At the herd level, the general immunity is also important and is influenced by both vaccine usage and vaccination protocols [14, 23–25]. Several studies have documented that piglets can become infected, as early as three days of age, despite the presence of maternally derived antibodies (MDAs). In addition MDAs may have unintended effects such as lack of seroconversion following infection and prolonged swIAV shedding [2, 3, 18, 26].

In Denmark, only inactivated swIAV vaccines are available, and they are mainly used in sows for clinical protection of piglets during the first weeks of life [27, 28]. Different studies have shown varying effects of different vaccines on the marked under field conditions [23, 29–36] probably due to differences in homology between the vaccine and herd strains and differences in vaccination protocols.

In this study, we aimed to investigate the viral dynamics, clinical signs, average daily weight gain and potential risk factors for swIAV detection such as serum antibody levels, use of cross-fostering and sow parity through longitudinal field studies performed in two Danish sow herds. In addition, in one herd (Herd 2), the effect of interventions such as change in vaccination protocol and selected management changes were evaluated.

## Methods

The study was part of a European collaborative project called “PIGIE” (Pig Influenza Genetics, Intervention and Epidemiology) funded by ICRAD 2821ERA24 [37], where six European countries (Germany, France, Spain, Italy, Denmark and Great Britain) investigated the infection dynamics of swIAV in sow herds.

### Ethics approval declarations

In accordance with the guidelines for good experimental practices (GEP) this study was conducted and approved by the Danish Animal Experimentation Council (protocol no. 2021-15-0201-01082).

### Sample size calculation

For the sample size calculations, we aimed to estimate the prevalence at a given time point; We assumed an overall incidence of 50%, in line with a previous Danish study [38], an allowable error of 12%, and a confidence level of 0.95. Adjusting for a population size of 680 piglets per batch this resulted in a sample size of 61 piglets [39]. Finally, a sample size of 60 piglets per batch was chosen.

### Study design and sample collection

The observational longitudinal field studies were conducted in two conventional Danish sow herds (Herd 1 and 2). The herds were sampled from November 2021 to June 2022, and sampling after the subsequent intervention in Herd 2 was performed from September 2022 to December 2022. The selection criteria for the herds were the presence of ≥ 400 sows, no swIAV vaccination of piglets and an endemic swIAV status.

Prior to the study start, a screening of the two herds was performed to confirm the endemic swIAV status and to identify the circulating swIAV subtype(s)/lineage(s). Twenty [20] nasal swabs were obtained from 5 pigs in four different age groups (week 2, 3, 5 and 6 weeks after farrowing) in each herd.

The nasal swabs were collected with sterile rayon swabs (Medical Wire, Corsham, UK). The swab was inserted and turned around in both nostrils of each pig. Afterwards, the swabs were placed in 2 ml Sigma Virocult media (Medical Wire) and kept at approximately 5 °C for a maximum of two days until nucleic acid extraction.

**Fig. 1:**
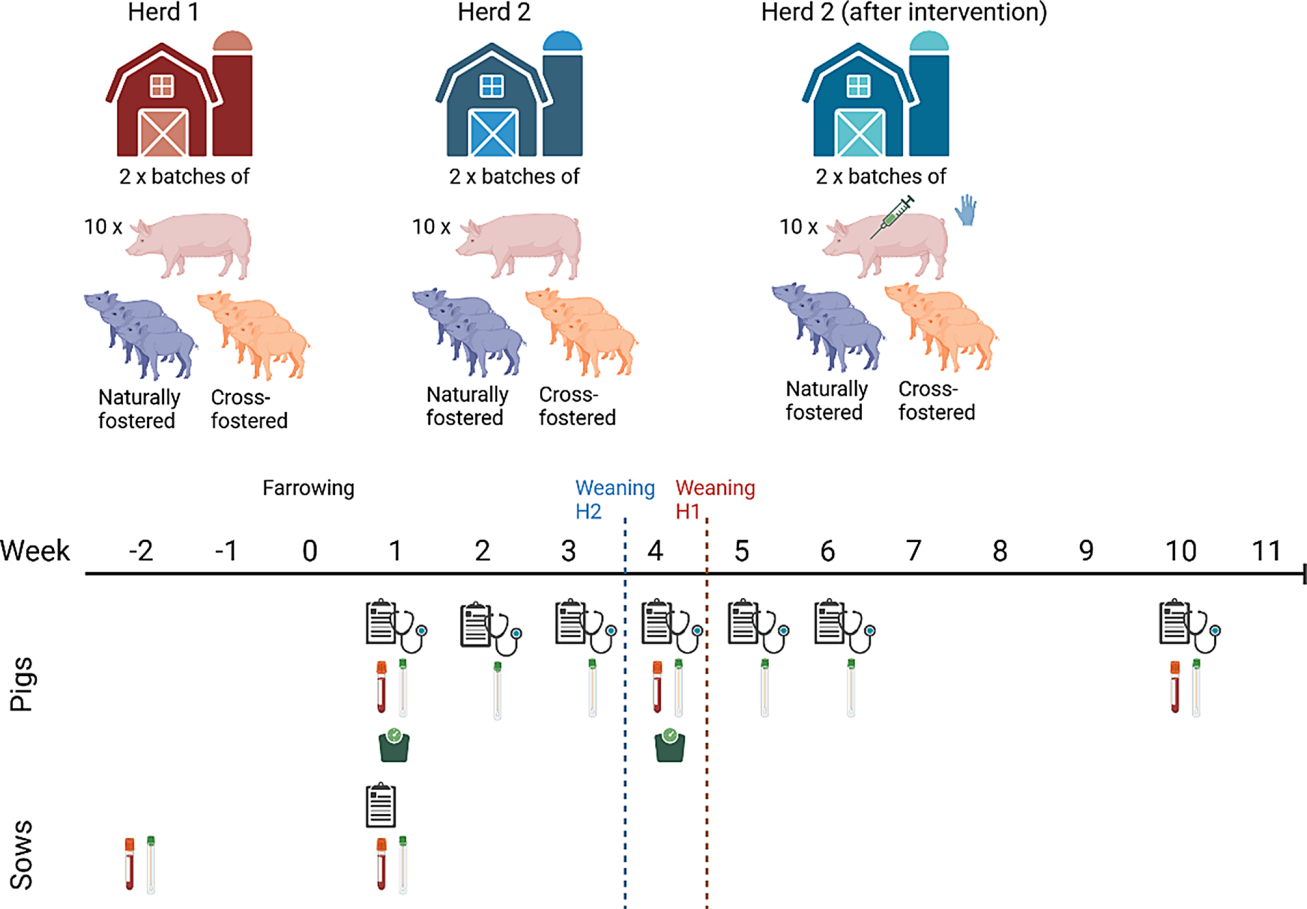
Overview of the study design. The figure illustrates the inclusion of two herds (Herd 1(red) and Herd 2(blue) with two consecutive batches of sows and their litters. In each herd, piglets were either naturally fostered (shown in blue) or cross-fostered (shown in orange). Herd 2 is shown both before and after the intervention, which consisted of pre-farrowing vaccination, hygiene and management changes. Sows were sampled two weeks before (-2 W) and one week after farrowing (W 1). Piglets were sampled weekly from week 1 (W 1) to week 6 (W 6) and again in week 10 (W 10/11), with samples consisting of nasal swabs and/or blood. Clinical signs were monitored at each sampling. Vertical dashed lines indicate the time of weaning in Herd 2 (blue) and Herd 1 (red). was recorded for the pens at each sampling. point in herd 1 (H1) and herd 2(H2). The red and blue dotted lines indicate time of weaning for Herd 1 (H1) and Herd 2 (H2)

In the study, two batches of pigs were sampled from each of the herds. From each batch, ten out of approx. 40 sows farrowing in the same week were included. From each of the ten sows six piglets of the litter (consisting of approx. 14 piglets) were included for sampling. To investigate if cross-fostering had a potential impact on the presence of swIAV in the individual pigs, three of the piglets were the sows’ own piglets (naturally fostered) and three were cross-fostered piglets. The naturally fostered piglets received blue earmarks, and the cross-fostered piglets received orange earmarks. Each earmark had a unique number. The sows were sampled twice; two weeks before and one week after farrowing and the piglets were sampled for six consecutive weeks (week 1–6) and at week 10 before exiting the nursery (Fig. 1).

#### Herd characteristics

Herd 1 was a farrow-to-nursery herd with 820 sows, divided into three farrowing units with a two-week batch production. The pigs were sold at 30 kg. In accordance with the Danish Specific Pathogen Free (SPF) system [40], the herd was declared positive for antibodies against *Mycoplasma hyopneumoniae (Myc)*, *Actinobacillus pleuropneumoniae 6* (Ap 6) and *Actinobacillus pleuropneumoniae 12 (Ap 12)*,* but were free of porcine reproductive and respiratory syndrome virus (PRRSV)*,* Ap 1–5 and 7–10*,* Brachyspira hyodysenteriae (B. hyo)*,* porcine atrophic rhinitis*,* mange and lice* [40]. The herd did not regularly vaccinate against swIAV, but it was decided by the herd veterinarian to mass vaccinate all sows, after including of the first batch of sows for this study. However, this was not regarded as an intervention in the study since the vaccine was not used according to the SPC as no booster was applied. The sows were vaccinated against swIAV with Respiporc Flu3 (CEVA Animal Health; France) [25]. The herd had an internal recruitment of gilts for breeding. When the herd used nurse sows, the piglets from the sows were moved to a baby unit before the actual weaning day. The herd used all in/all out when moving pigs between the farrowing unit and the nursery unit. At weaning, the piglets were moved to the nursery unit “litter wise” so that three-four litters were weaned into one pen. The production parameters resembled the national average [41]. This herd followed a biosecurity protocol requiring personnel to change clothes and shoes before returning to younger piglets/pigs. For example, if assistance was needed in the nursery unit and staff had to go back to the farrowing unit, they would change their clothes, etc.

Herd 2 was a farrow-to-nursery herd with 800 sows, divided into five farrowing units and weekly batch production, with a continuous flow in the farrowing unit, meaning that sows from different weekly batches were mixed within the unit. Early weaned piglets stayed without a sow until the remaining batch were weaned. The smallest piglets were weaned separately into a baby unit and was later moved to a nursery unit. Two-thirds of the pigs were sold at 30 kg, while the remaining pigs were kept until slaughter in a finisher stable near the gilt unit. In accordance with the Danish SPF system, the herd was declared positive for antibodies against Myc and Ap12, but were free of PRRSV, *Ap 1–10*,* B. hyo*,* porcine atrophic rhinitis*,* mange* and *lice* [40]. Gilts were bought from an external supplier every third month and housed in the finisher unit until insemination. Sows were vaccinated four times a year with Respiporc FLU3 (CEVA Animal Health, France) [27] and gilts were vaccinated with both Respiporc Flu3 and Respiporc FLUpan (H1N1pdm) (CEVA Animal Health, France) [42] upon arrival in the herd. The production parameters resembled the national average [41].

#### The intervention study in herd 2

After the longitudinal studies, described above, were conducted in the two herds, an intervention study was performed in Herd 2. The collection and analyses of the samples was as described for the longitudinal study (Fig. 1). The interventions included pre-farrowing vaccination two weeks before farrowing of all sows in both weekly batches wherefrom the 20 sows were selected for sampling with both Respiporc FLU3 [27] and Respiporc FLUpan [42]. Litter equalization was performed within the first 24 h after birth to allow colostrum intake. After this procedure, piglets were not allowed to be moved between litters. Additionally, at weaning, the litters sampled in this study were weaned litter-wise to the same pens. The herd personnel started using disinfectants of hands between different units, and if tools were moved between units, they were also washed and disinfected. It should be noted that the two farrowing units that housed the two batches of the intervention study was located centrally in the herd and used for passage between the baby units and sick pens.

### Nasal swabs

#### RNA extraction from pooled nasal swabs

Prior to RNA extraction, the nasal swabs were vortexed to transfer the biological material to the Sigma Virocult media, and 100 µL per sample was used for pooling.

The nasal swabs were pooled per litter (six nasal swabs per pool). The RNA was extracted using the RNeasy Mini Kit (QIAGEN) automated on the QIAcube Connect (QIAGEN) according to the manufacturer’s instructions with a swIAV isolate as positive RNA extraction control and nuclease-free water as a negative control. The RNA was stored at − 80 °C until further analysis.

#### Real-time reverse transcription PCR (real-time RT‒PCR) of pooled nasal swabs

A previously published real-time RT‒PCR assay targeting the matrix gene of swIAV [43] was used to determine if a pool was swIAV positive. All PCR tests were run on the Rotor-Gene Q (QIAGEN) PCR machine and were analysed in duplicates and with a positive swIAV control and a negative control in each run. All samples with a cycle threshold (Ct) value less than 36 were considered positive. If a pool was positive for swIAV, the individual samples of that pool were tested as described in the following section.

#### Nucleic acid extraction of individual samples from swIAV-positive pools

For each individual nasal swab sample, 200 µL was used for extraction of total nucleic acids. Positive controls (swIAV serving as control for RNA extraction and PCV2 serving as control for DNA extraction) and a negative control were included in all extractions. Total nucleic acid was extracted on the QIAcube HT (QIAGEN) with the Cador pathogen 96 QIAcube HT Kit (IndiSpin^®^ QIAcube^®^ HT Pathogen Kit, QIAGEN Hilden, Germany) according to the manufacturer’s instructions. After extraction, the nucleic acids were stored at -80 °C until further analysis.

#### Reverse transcription and pre-amplification

Prior to the final high-throughput real-time PCR analysis, the extracted individual nasal swabs were subjected to reverse transcription and pre-amplification.

For the RNA targets, reverse transcription and pre-amplification were performed in a final volume of 15 µL using the AgPath-ID one-step RT‒PCR reagents kit (Applied Biosystems, Thermo Fisher Scientific, Waltham, MA, USA). For the DNA targets, pre-amplification was performed in a final volume of 10 µL using the TaqMan PreAmp master mix (Applied Biosystems). The details of both reactions can be found in Additional file 1.

#### RHigh-throughput real-time PCR

For high-throughput real-time PCR analysis, individual nasal swabs were analysed for the presence of swIAV specific genes: M, H1pdm, H1av, H3hu, H3sw, N1pdm, N1, N2sw and N2hu. The primer and probe sequences for each of target gene, which are described and validated in previous studies [44, 45] are listed in Additional file 2 [6, 44–48].

For the high-throughput real-time PCR analysis, a 192.24 DA IFC chip (Standard BioTools, South San Francisco, USA) and the BioMark HD platform (Standard BioTools) were used, and the PCR analysis was performed as previously described [46].

#### Virus isolation in MDCK cells

A selection of the nasal swabs with high viral loads in the high-throughput real-time PCR analysis and samples that represented all circulating strains in the two herds were selected for virus isolation, and they were subsequently analysed in the hemagglutination inhibition (HI) test. This was done as described in a previous study by sterile filtrations of nasal swabs being inoculated onto MDCK cells [48].

#### Whole-genome sequencing of the herd strains and data analysis

Virus isolates and individual SwIAV positive nasal swabs collected from the screening in the two herds and during the longitudinal studies and the intervention study were subjected to conventional PCR using the universal influenza primers as previously described [6]. Thereafter whole genome sequencing was performed using the Illumina MiSeq platform and consensus sequences of each segment were generated the subtype and genotype determined as previously described [48].

Additionally, the HA clade was determined using the swine H1 global classification tool at https://www.bv-brc.org/app/SubspeciesClassification. The identity of the HA protein to the two matching vaccine strains currently available in Denmark (Respiporc FLUpan H1N1: A/Jena/VI5258/2009 (H1N1) and Respiporc FLU3: Haselünne/IDT2617/2003 (H1N1) [27, 42]) was evaluated by alignments to the translated amino acid sequence of the herds strains.

### Clinical signs

At each sampling, the ear-marked pigs were clinically examined. The clinical signs recorded included dyspnoea, lacrimal discharge, nasal discharge (s = serous, m = mucus, p = purulent), conjunctivitis, the body condition score (1–4 (1 = contours of spine and ribs are clearly visible, 2 = contours of spine and ribs are visible, 3 = contours of spine can be seen, while the ribs are not evident and 4 = contours of spine and ribs are not evident) and it was noted if the pig had any other illness or was treated with antibiotics. At weeks 1 and 4, the pigs were also individually weighed.

Every pen with earmarked pigs, both in the farrowing unit and in the nursery unit, had a coughing index calculated at each sampling. The method was based on a previous study, counting the number of coughs/sneezes for three minutes and the number of pigs in the pen [49].

### Serum samples

Blood samples were collected from the included sows two weeks before- and one week after farrowing. Furthermore, blood samples were collected from the ear-marked pigs at week 1, 4 and before they left the nursery unit (week 10–12). Blood samples were collected from *vena jugularis* of the sows and *vena cava cranialis* of the piglets and stored in vacutainer serum tubes (Becton – Dickinson, Denmark) at approximately 5 °C for a maximum of two days until they were centrifuged at 3000 rpm for ten minutes, after which the serum was frozen at -20 °C until further analysis.

#### Enzyme-linked immunosorbent assay (ELISA) test

Antibodies against influenza A virus were measured using an indirect ELISA targeting the nucleoprotein (NP) (ID Screen Influenza A Nucleoprotein Swine Indirect, IDvet, Grabels, France) following the manufactures protocol [50]. The S/P ratios were converted to serum antibody titers using the manufacturer’s calculation method, and titers > 1053 corresponding to the S/P ratio > 0.4 were considered as positive.

#### Hemagglutination-inhibition (HI) test

Serum samples from five sows and their six piglets (*n* = 30) were selected from each herd for the HI-test. The inactivation of the pigs serum, hemagglutination (HA) test and the following HI-test was performed as described in a previous study, with guinea pig blood used in the RBC step [51].

### Statistical analysis

Data records were initially registered in Microsoft Excel 2016 and subsequently exported to GraphPad Prism version 9.5.1 for Windows (GraphPad Software, San Diego, California, USA; www.graphpad.com) and R version 4.2.2 for Windows (The Comprehensive R Archive Network (r-project.org)) for data management and analysis [52]. A significance level of *p* < 0.05 was used for all analyses.

Incidence risk and rate were calculated to assess the development in new cases of influenza shedding piglets over time [53].

Statistical models were used to quantify the risk factors for the shedding of influenza at a certain time point, by means of generalized linear mixed models with binomial outcome (influenza shedding yes/no). Due to the hierarchical structure of data; where time periods were clustered within piglets, clustered within sows, clustered within batches, clustered within herds, random effects were included in all models to control for this potential clustering. Univariable analyses were carried out for each risk factor of interest, as an initial screening. Only risk factors sufficiently associated with the outcome of interest (*p* < 0.20 in the univariable analysis) proceeded for inclusion in full model of the multivariable analysis. A backward elimination of the full model was subsequently carried based on likelihood ratio-test and a significance level of 0.05, resulting in the final model. The packages EpiR [54] were used for the estimation of prevalence, ggplot2 [55] for the illustration of plots, lme4 [56] to fit the generalized linear models [52].

To investigate differences in swIAV ELISA antibody titers and HI titers between swIAV positive and negative pigs, cross-fostered and natural fostered, increase or decrease in titer(response) after IAV infection an unpaired, non-parametric Mann-Whitney U test were applied for each herd, since the data did not pass the normality tests in GraphPad Prism.

A correlation analysis was performed in GraphPad Prism to compare the HI titer from the selected pigs in each herd with the corresponding ELISA antibody titer using Spearman’s correlation analysis in GraphPad Prism. A similar analysis was performed to compare the ELISA antibody titer of the sows two weeks before farrowing and the piglets at week 1 in each herd.

## Results

### Screening for SwIAV in the two herds

The screening confirmed the enzootic status of swIAV in both herds as 4/5 and 3/5 pools tested positive for swIAV in Herd 1 and Herd 2, respectively. Whole genome sequencing revealed that Herd 1 had an H1N2 subtype circulating (H1C.2.4N2G), with the HA gene belonging to the 1 C.2.4 clade, the NA gene of the Ghent N2 lineage and an internal gene cassette of Eurasian avian-like H1N1 origin (AAAAAA) (NCBI GenBank accession no.: PV660160-PV660167). Similarly, an H1N2 (H1C.2.4N2G) was circulating in Herd 2 with similar origins of the HA and NA genes but with an internal gene cassette of H1N1pdm09 origin (PPPPPP) (NCBI GenBank accession no.: PV660224-PV660231). The HA protein of the two herds strains shared 89% and 92% to the vaccine strain of 1 C.2 origin in Respiporc FLU3 (Haselünne/IDT2617/2003).

### Longitudinal field studies

#### Detection of swIAV

**Fig. 2:**
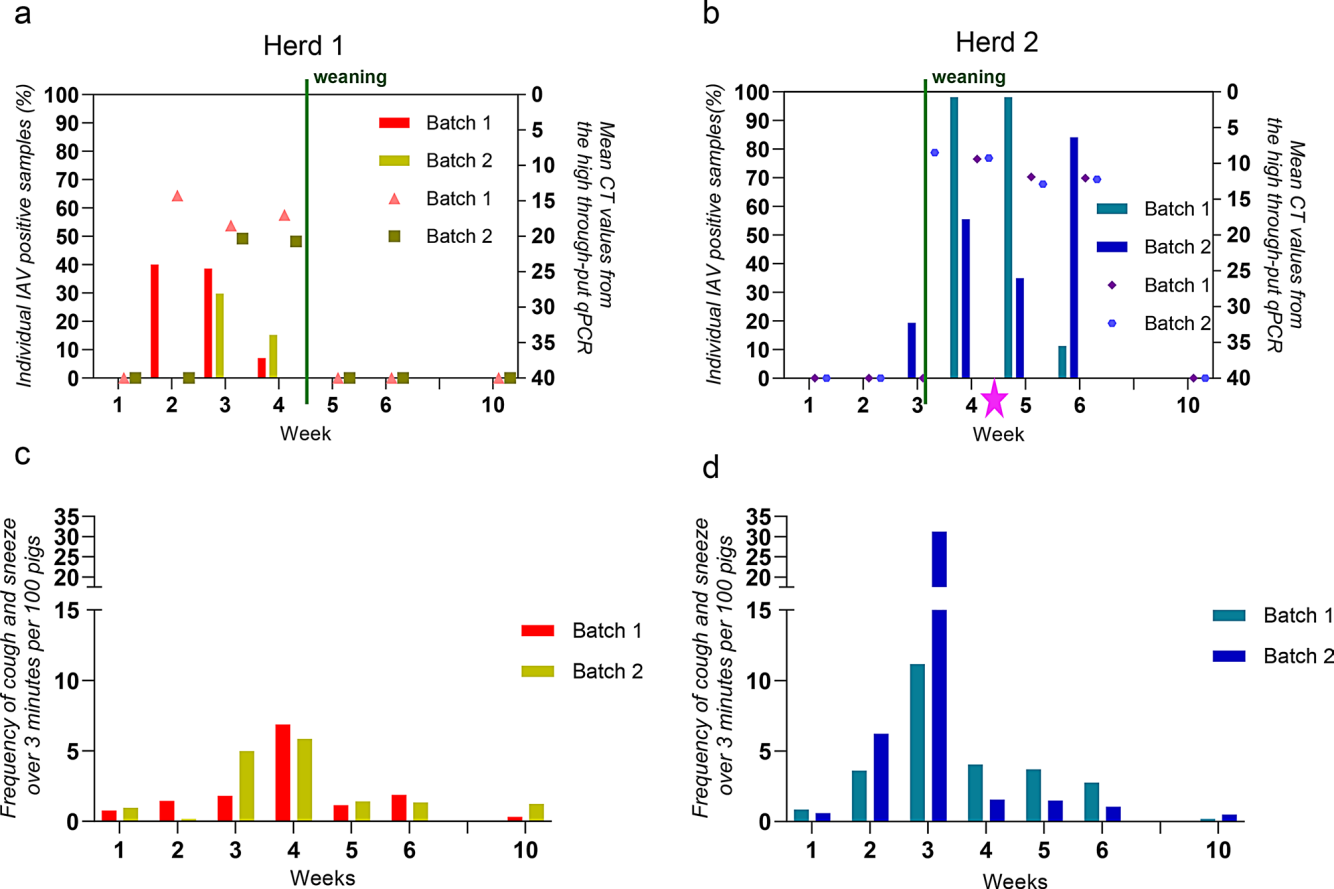
Viral shedding and cough and sneezes. Figure **a** and **b**. Prevalence of high-throughput RT-PCR positive pigs (bars) and the average CT value (triangles, rhombus, hexagon, and squares)) SIV M-gen) for each sampling point and batch. Green line indicate weaning. The pink star indicates an introduction of a new subtype H1N1pdm09 in Herd 2. Figure **c** and **d**. The calculated coughing index. The index is calculated over 3 min where coughs and sneezes are counted, then divided by the number of piglets in the pens

In total, 228 pigs were included in the study (Herd 1 (*n* = 108) and Herd 2 (*n* = 120)). Overall, 64% and 97% of the individually included pigs in Herd 1 and Herd 2, respectively tested positive for swIAV at least once during the study period. In Herd 1, swIAV shedding occurred between week 2 and 4 in both batches. For batch 1, 40% of the pigs were positive in week 2, 39% in week 3 and 7% in week 3. In batch 2, 30% were positive in week 2, and 15% were positive in week 3 (Fig. 2a). No swIAV positive pigs was observed in the nursery unit in any of the batches (week 5, 6 and 10). In Herd 2, swIAV positive pigs occurred in the farrowing unit (at week 3–4) and continued in the nursery unit. For batch 1, 98%, 98%, and 11% were positive in week 4, 5 and 6, respectively. In batch 2,19% were positive in week 3.56% in week 4.35% in week 5 and 84% in week 6 (Fig. 2b).

In Herd 1, 57.4% [62] pigs tested positive for the H1C.2.4N2G subtype at one sampling and seven pigs tested positive for two consecutive weeks, indicating prolonged shedding for more than one week (Additional file 3).

In Herd 2, eight (6.7%) pigs tested positive for the H1C.2.4N2G subtype at one sampling, six pigs at two consecutive samplings, and one pig at three consecutive samplings. The pandemic H1N1pdm09 subtype was introduced in Herd 2 during week 4 (batch 1) and week 5 (batch 2). A total of 14 pigs (11.67%) tested positive for this subtype at a one sampling. Across the study period, 87 (72.50%) pigs in Herd 2 were infected with both the H1C.2.4N2G and the H1N1pdm09 strains. Among them, 31 were first infected with the H1C.2.4N2G subtype and later acquired the H1N1pdm09 subtype. Of these, nine pigs tested negative for one to two samplings before the new infection, while 22 were infected with H1N1pdm09 at the sampling immediately following a positive test for H1C.2.4N2G. The remaining 30 pigs (of the 87 infected pigs in Herd 2) either had a mixed infection or tested positive only in the M-gene, preventing subtype determination (Additional file 3).

#### Clinical signs

An increase in the coughing index was observed at the same time as most of the piglets tested positive for swIAV in both herds (Fig. 2c and d). The only clinical sign significantly correlated with the detection of swIAV in nasal swabs was nasal discharge. In the combined univariable analysis of the two herds, nasal discharge was strongly associated with swIAV shedding (*p* = 0.002), with an odds ratio of 2.36, which means that pigs with swIAV are 2.63 more likely to have nasal discharge than pigs without swIAV. No significant difference was found between dyspnoea, conjunctivitis or tear discharge and swIAV shedding.

#### Risk factors

Herd, as an effect, influenced the number of pigs that tested swIAV positive(*p* < 0.05), which reflects the difference in swIAV prevalence in the two herds. The number of swIAV positive piglets was not significantly correlated to sow parity. Similarly, the number of swIAV positive pigs did not significantly differ between the sows’ own pigs and the cross-fostered pigs.

Weight gain was compared between swIAV-infected and non-infected pigs in Herd 1, the only herd where not all piglets tested positive for swIAV and where the swIAV shedding occurred between the two weights (week 1 and 4). No significant different difference in weight gain was observed between infected and non-infected pigs.

#### Incidence risk

For herd 1 the incidence risk was 0.62 that means that the individual pig had a risk at 62% of getting IAV during the time in the farrowing unit. At batch level, it was 78% and 42% risk for batch 1 and batch 2, respectively. For herd 2 the incidence risk was 0.97, meaning that the individual pig had a risk at 97% to getting IAV at some point during the study, while at batch level it was 100% for batch 1 and 94% for batch 2.

In this study, we had a sampling period of 12 weeks. In herd 1, we investigated 108 pigs and in herd 2, we investigated 120 pigs. In herd 1, 67 pigs were shedding IAV, while 116 pigs were shedding IAV in herd 2, resulting in the incidence rate being five and eight new IAV shedding cases per 100 pigs per week for herd 1 and herd 2, respectively.

#### Whole-genome sequencing analysis of the herds strains

The herd strain circulating in Herd 1 during the study period was the same as that recorded in the screening of the herd with a minor level of single nucleotide polymorphisms (SNPs) introduced (NCBI GenBank accession no.: PV660168-PV660183). In Herd 2, similarly, the same H1N2 strain as during the screening was observed in the study period (NCBI GenBank accession no.: PV660184-PV660207). However, a newly introduced H1N1pdm09 (clade 1 A.3.3.2) with all genes of H1N1pdm09 origin apart from the NS gene, which was of Eurasian avian-like H1N1 origin (PPPPPA) was also detected (NCBI GenBank accession no.: PV660208-PV660223). The HA amino acid identity to the H1N1pdm09 vaccine strain of Respiporc FLUpan H1N1 was 92%.

#### SwIAV antibodies

**Fig. 3:**
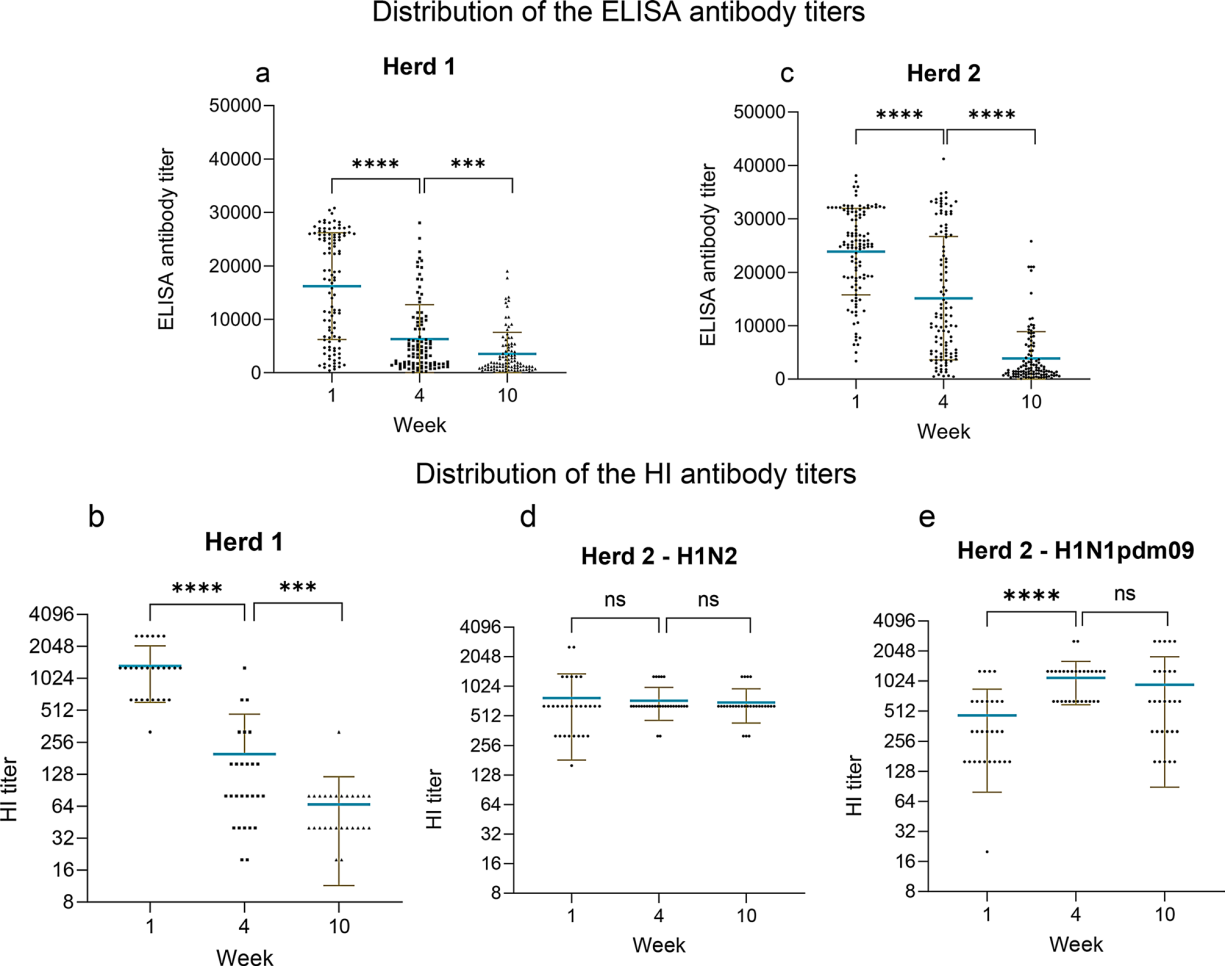
Comparison of the ELISA antibody titers and the HI antibody titers in pigs. Each point represents a titer of an individual pig. The brown lines indicate error bars and the blue horizontal line indicates the mean titer. Significant differences between week one and four and between week 4 and 10 are indicated by * (*** = p = < 0.001: ****= p = < 0.0001 and NS = no significant)

In Herd 1, 100% and 75% of the sows were seropositive two weeks before farrowing in the two batches, respectively and 100% in both batches were seropositive one week after farrowing. Among the piglets, 95% and 100% (week 1), 79% and 93% (week 4), and 64% and 82% (weeks 10–12) were seropositive in batch 1 and 2, respectively. For herd 2, 100% of the sows were seropositive both two weeks before and one week after farrowing Among the piglets, 100% and 100% (week 1), 88% and 100% (week 4) and 40% and 88% (week 10–12) were seropositive in batch 1 and 2, respectively. To investigate the swIAV ELISA antibody titers among the piglets at the different samplings, a comparison was made between each sampling time (Fig. 3a and c). In Herd 1, a significant difference was found between each week from a mean of 16,245 in week 1 to 6340 in week 4 (*P* < 0.001) and to a mean of 3535 in week 10 (*p* < 0.001) showing a decline in the mean antibody titer over time. The same pattern was observed in Herd 2 with a significant decrease in antibody titer over time with a mean of 23,893 in week 1, 15,157 in week 4 and 3858 in week 10 (*p* < 0.001).

In total, serum samples from five randomly selected sows and 30 of their piglets (six per sow) were tested in the HI-test against the herd specific viruses. To compare the HI titers between the weeks a Mann Witney t-test was performed. In Herd 1, a significant difference was found in HI titer between each week with a mean titer of 1339 in week 1, 198 in week 4 and 66 in week 10 (*p* < 0.001) (Fig. 3b). In Herd 2, there was only a significant increase in HI titer for the H1N1pdm09 subtype between week 1 and week 4, with an HI-titer of 464 in week 1 versus 1097 in week 4 (*p* < 0.001). No increase or decrease was observed from weeks 4 to 10/12. The HI antibody titer against the H1C.2.4N2G remained stable over time with no significant differences between the weeks (Fig. 3d and e).

#### Differences in antibody titers between natural fostered and cross-fostered piglets

**Fig. 4:**
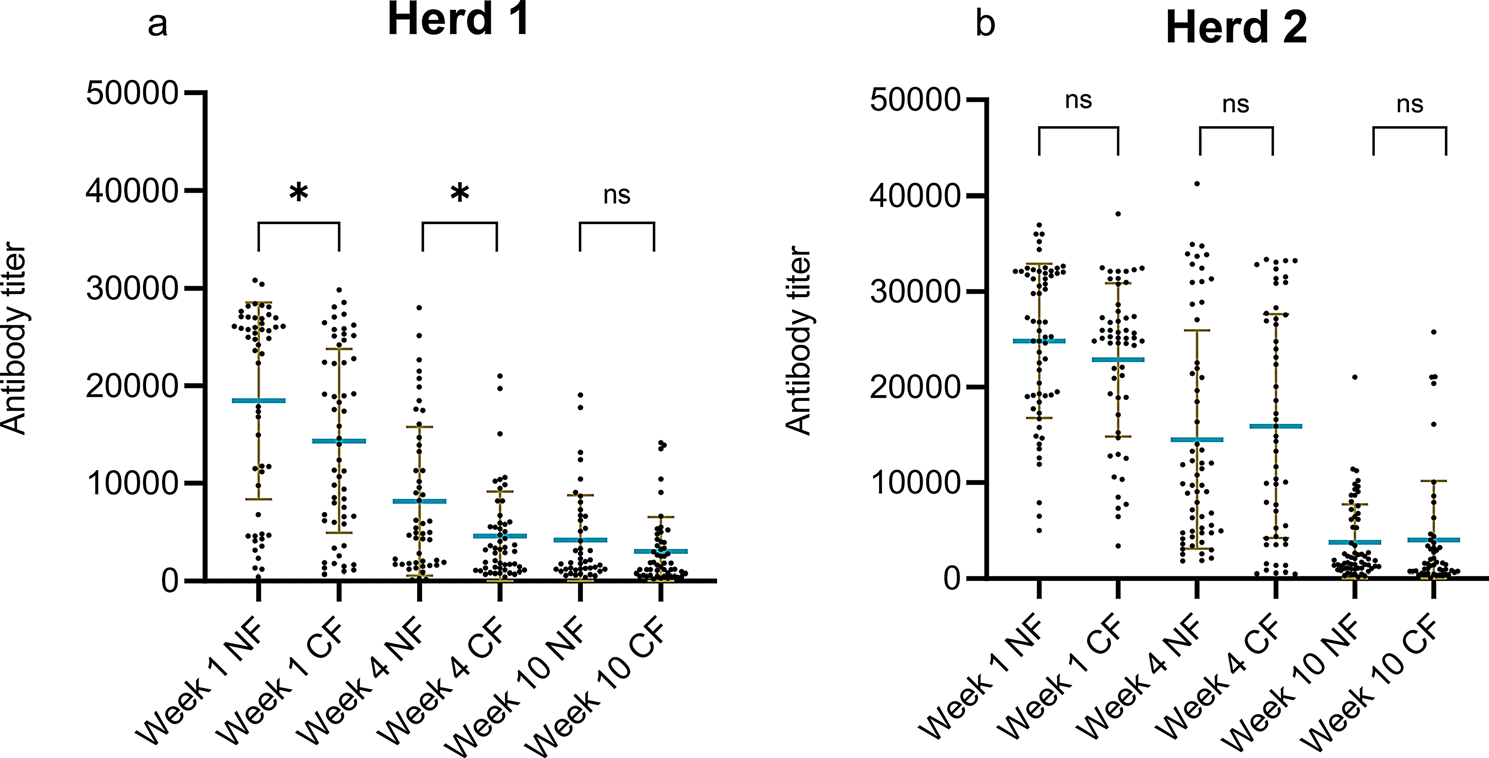
Comparison of the ELISA antibody titers of the natural fostered (NF) or cross fostered(CF) pigs. Each point represents a titer of the individual pigs. The brown lines indicate error bars and the blue horizontal line indicates the mean titer. The p-value between CF and NF in weeks 1, 4 and 10 were *p* = 0.0197, *p* = 0.0158 and *p* = 0.1063 in Herd 1. In Herd 2 the p-value between CF and NF in week 1, 4 and 10, were *p* = 0.1699, *p* = 0.7526 and *p* = 0.1525

The differences in ELISA (Fig. 4a and b) and HI (Additional file 4) antibody titers between cross-fostered and natural fostered pigs, were compared using the Mann-Whitney t-test. In Herd 1, a significant difference was observed, with cross-fostered piglets having a lower mean ELISA titer at both week 1 and week 4. Week 1 the mean titer was 14,341 in cross-fostered piglets versus 18,546 in naturally fostered piglets (*p* < 0.01) and in week 4 these titers were 4569 versus 8157 (*p* < 0.01). A similar significant difference in the cross-fostered piglets at week 1 and week 4 was observed in the HI-test, with an HI-titer of 1009 for cross-fostered and 1646 for naturally fostered in week 1 versus 157 for cross-fostered and 237 for naturally fostered in week 4 (*p* < 0.01). In Herd 2, there were no significant differences in antibody titers between the cross-fostered and naturally fostered piglets in either the ELISA or HI test.

#### Impact of cross-fostering on maternal antibody transfer to piglets

**Fig. 5:**
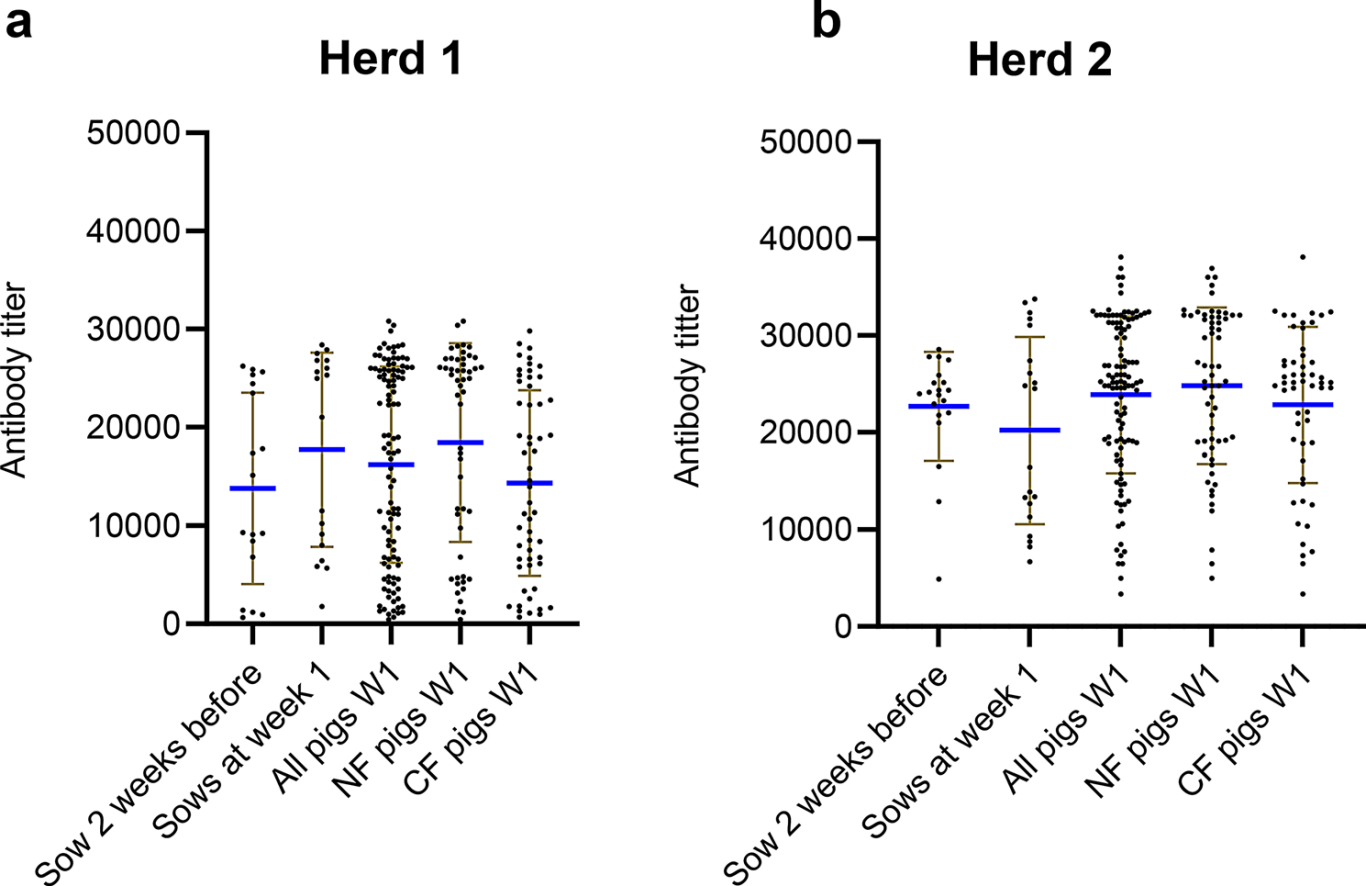
Comparison of the sows ELISA antibody titer two weeks before farrowing and the serum ELISA antibody levels at week 1 in herd 1 (**a**) and 2 (**b**) in all pigs, natural fostered (NF) pigs and cross fostered (CF)) pigs, respectively. Each point represents the titer of each individual pig. The brown lines indicate error bars and the blue horizontal line indicates the mean titer

To further evaluate that cross-fostering can have an impact on the uptake of MDA, the week 1 antibody titers of all pigs independent of their fostering, natural fostered pigs and cross-fostered pigs, respectively were compared with the antibody titer (two weeks before farrowing) of the sow from which they were raised. The mean and individual ELISA antibody titers for sows (before and after farrowing) and piglets (at week 1) are shown in Fig. 5. To assess the impact of sow antibody titers on piglet antibody levels at week 1, a Spearman correlation analysis was performed using GraphPad Prism. Piglets were categorized into three groups: naturally fostered, cross-fostered, and a combined group.

In Herd 1 and Herd 2, a significant but weak correlation (*r* = 0.19, *p* < 0.05) was found between the combined group’s antibody titers and the sows antibody titer two weeks before farrowing, suggesting some increase in level of MDA transfer from the sows to their offspring, but with considerable variability. However, in the natural fostered group, a stronger correlation was observed (*r* = 0.43–0.45, *p* < 0.001) indicating a more consistent transfer of MDA, when the piglets remained with their biological mother. In contrast, the cross-fostered group showed no significant (*p* > 0.05), emphasizing that moving piglets potentially disrupt MDA transfer.

#### Impact of MDA on protection and immune response after swIAV infection

To investigate if the level of MDA uptake at one week of age was a determinant for the pig subsequently becoming infected with swIAV, the average ELISA antibody titer at week 1 of the infected and non-infected piglets at some point during the study were compared for Herd 1. This analysis was not performed in Herd 2 as very few piglets did not become infected. In Herd 1, no significant difference was observed in the ELISA titers between pigs that became infected and uninfected pigs when comparing their ELISA antibody titer at week 1 (*p* > 0.05).

**Fig. 6:**
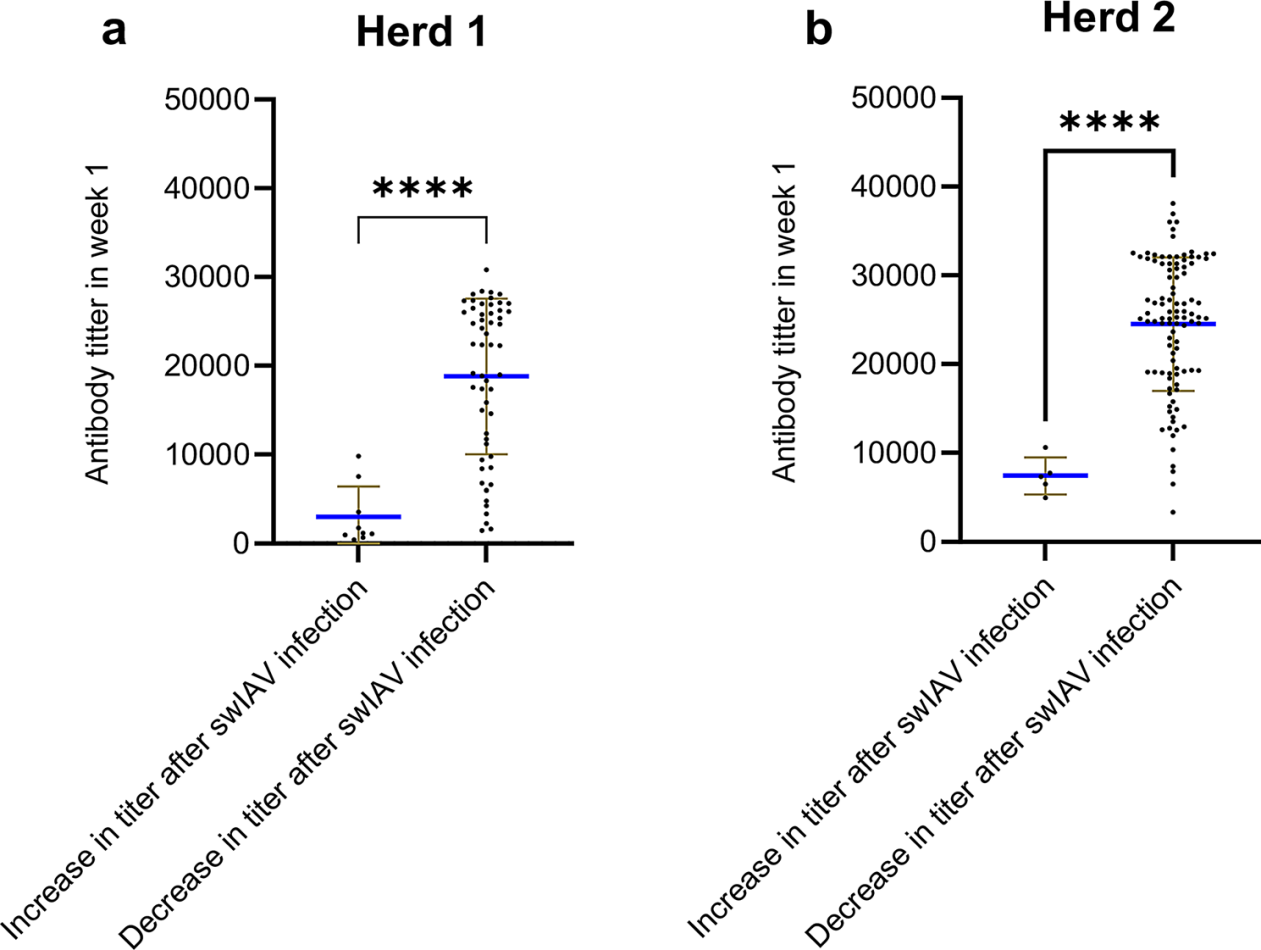
ELISA antibody titers between pigs that develop a serological antibody response after swIAV infection and pigs with a decrease after swIAV infection in both Herd 1(**a**) and Herd 2(**b**). Each point represents the titer of an individual pig. The brown lines indicate error bars, and the blue horizontal line indicates the mean titer. Significant difference indicated with **** = *p* < 0.0001

To investigate if the ELISA antibody titer increased or decreased following swIAV infection and if this was determined by the MDA level at week 1 the average week 1 titers of the piglets experiencing an increase or decrease in titer after infection, were compared. In total, nine and five pigs had an increase in antibody titer after swIAV infection in Herd 1 and 2, respectively. On the contrary, 55 and 101 pigs had a decrease in antibody titer following swIAV infection in Herd 1 and Herd 2, respectively (Fig. 6). Interestingly, in both Herd 1 and 2, a significant higher (*p* < 0.01) mean ELISA titers at week 1 (3002 vs. 18800 in Herd 1 and 7420 vs. 24503 in Herd 2) were observed in the pigs that showed a decrease in antibody titer after swIAV infection.

#### Correlation between ELISA antibody titers and HI titers differs between herds

To examine the correlation between swIAV ELISA and HI test results, a Spearman correlation analysis was conducted using GraphPad Prism. The analysis included all HI titers for the H1C.2.4N2G and H1N1pdm09 strains, along with their corresponding ELISA antibody titers in each herd. In Herd 1, a moderate positive correlation was observed between ELISA titers and HI titers for the H1C.2.4N2G subtype (*r* = 0.4545, *p* < 0.001), suggesting a general alignment between the two antibody detection methods for this subtype. In Herd 2, no significant correlation was found between ELISA titers and HI titers for either strain (H1C.2.4N2G *p* > 0.05, H1N1pdm09 *p* > 0.05).

### The intervention study

#### Detection of swIAV

**Fig. 7:**
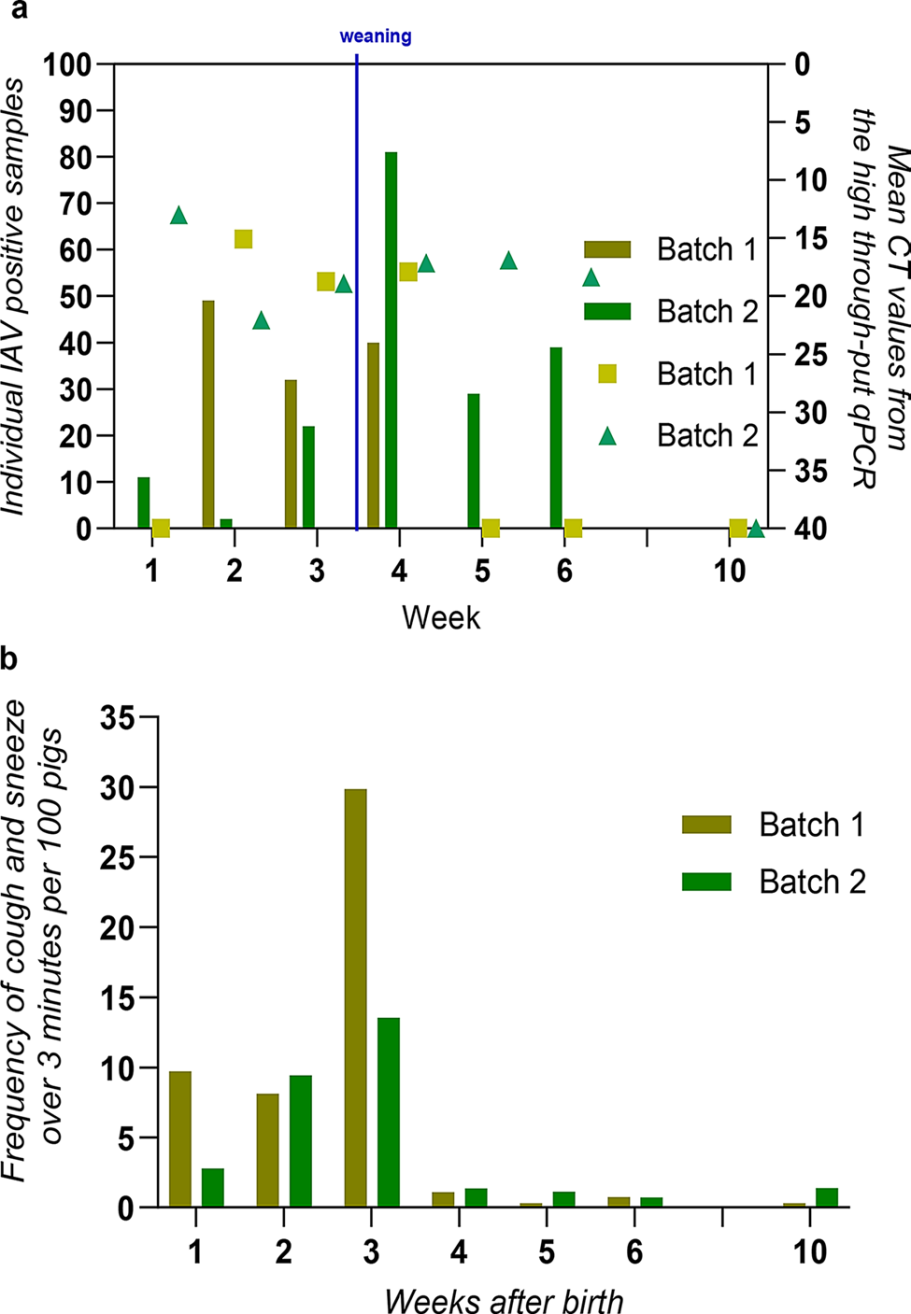
Overview of viral shedding and cough and sneezes in the intervention study. Figure **a** illustrates the viral shedding in each herd, both with the prevalence of positive pigs, showed with bars, and the average CT-value, showed with triangles, rhombus, hexagon, and squares, (SIV M-gen) from the high throughput qPCR at the given sampling point for each of the batches that were followed. The weaning time is marked with a blue line. The calculated coughing index are shown in figure **b**. The index is calculated over 3 min where coughs and sneezes are counted, then divided with the number of piglets in the pens

In the intervention study, 108 pigs were followed in two batches from farrowing to the end of the nursery unit from September to December 2022. In total, 79.6% of the pigs tested positive for swIAV at least once during the study, representing a significant 17% decrease compared to the first study in Herd 2 (*p* < 0.001). In batch 1, virus shedding occurred from week 2 to week 4 (49% in week 2, 32% in week 3 and 40% in week 4). In batch 2, swIAV shedding was observed from week 1 to week 6 (11%, 2%, 22%, 81%, 29%, and 39% were positive in week 1–6, respectively) (Fig. 7a). Additionally, the results showed that several pigs tested positive for swIAV at minimum two consecutive samplings, with 30.6% shedding for two consecutive weeks, and 12.0% for three consecutive weeks (Table 1, Additional file 3). The proportion of pigs with ≥ 2 weeks of consecutive shedding was significantly reduced by 52% compared to the first study in Herd 2 (*p* < 0.001). Only one subtype, the pandemic H1N1pdm09 (NCBI GenBank accession no.: PV660232-PV660247), similar to that detected during the longitudinal study, was observed.

**Table 1:** Number of pigs with consecutive weeks of viral shedding in the intervention study. Since only one subtype(H1N1pdm09) were found in the intervention study, all samples only positive in the SwIAV M-gen was suggested as positive for H1N1pdm09

Weeks of viral shedding	Herd 2
0	22
1	40
2	33
3	13
4	0

In the intervention study, the weekly incidence risk remained around 30% during the first six weeks. In contrast, the longitudinal field study showed an incidence risk of up to 65% during the four weeks when swIAV infection occurred. Overall, the intervention study exhibited a lower incidence. However, swIAV was detected over a longer period.

#### Clinical signs and risk factors

Similar to what was observed in the longitudinal study in Herd 2, an increase in the coughing index coincided with a peak in swIAV shedding (both the number of swIAV-positive pigs and the highest viral load) (Figs. 7b). There was a tendency that nasal discharge was associated with swIAV shedding (*p* > 0.06). No significant difference was found between dyspnoea, conjunctivitis, tear discharge and swIAV shedding.

Similar to the longitudinal study, there were no significant differences in swIAV shedding related to cross-fostering (*p* > 0.05) or among sow parities (*p* > 0.05).

#### Detection of antibodies

**Fig. 8:**
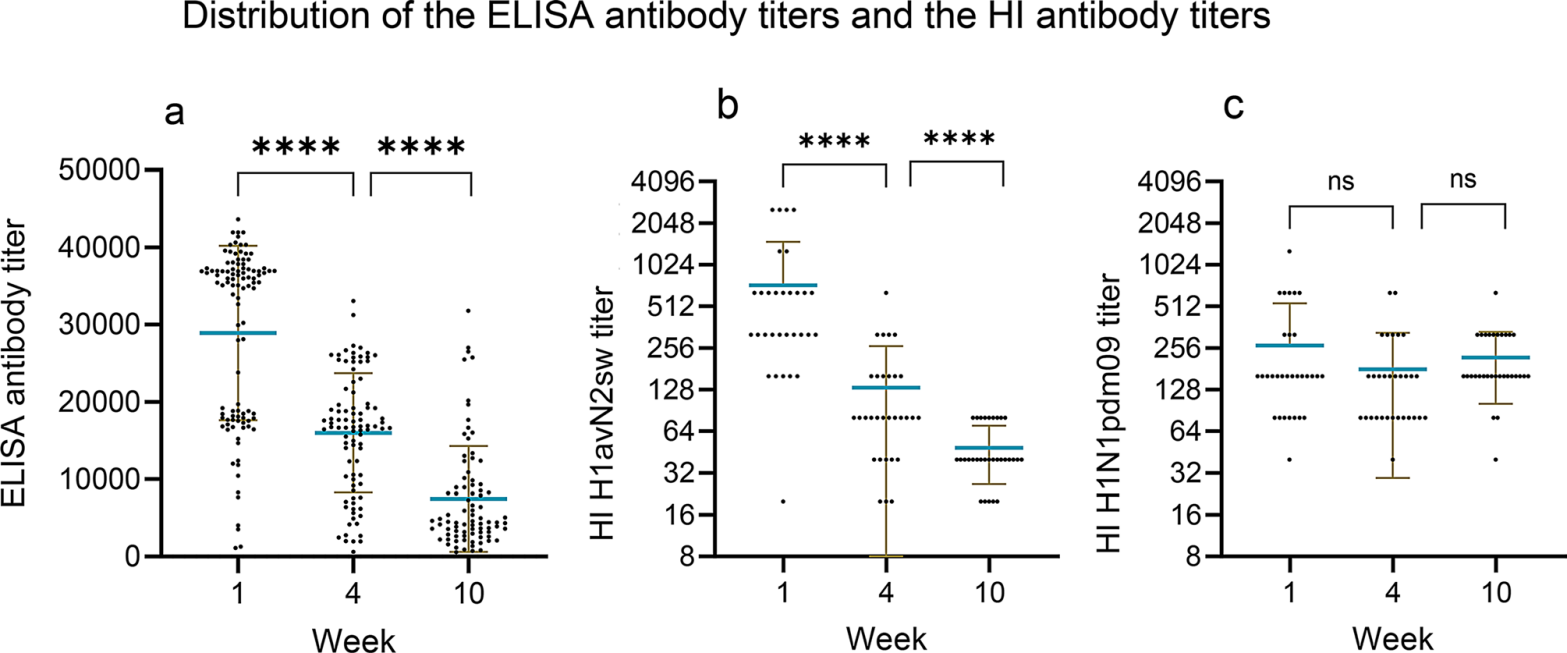
Comparison of the ELISA antibody titers (**a**), and the HI antibody titers among the piglets during the study (**b** and **c**). Each point represents a titer of an individual pig. The brown lines indicate error bars and the blue horizontal line indicates the mean titer. The ELISA antibody titer p-value between week 4 and 1 and week 10 and 4 was p = < 0.001. There was no significant difference between HI H1N1pdm09 titers at the sampling points (*p* = 0.0987 W1-4 and *p* = 0.2077 W4-10). For the HI H1avN2sw subtype, there was a significant difference both between weeks 1 and 4, and 4 and 10 (p = < 0.0001)

As mentioned, the included sows were vaccinated with Respiporc FLUpan and Respiporc FLU3 [27, 42] two weeks before farrowing. The ELISA results revealed that all sows had antibodies two weeks before farrowing and at week 1. Correspondingly, all the pigs had antibodies against swIAV in week 1 (100%) and almost all in week 4 (97–100%), and in week 10–12 (90–97%). Serum samples of six sows and their pigs (*n* = 31) were also subjected to HI-test, with the specific herd strains of swIAV (H1C.2.4N2G and H1N1pdm09). To investigate the swIAV ELISA antibody titers among the piglets at the different samplings, a comparison was made between each sampling (Fig. 8a). A significant difference was found between each week (*P* < 0.01) with a decline in the mean antibody titer over time from 28,900 in week 1 to 15,982 in week 4 and 7454 in week 10.

To compare the HI titers among the pigs between the samples, a comparison was made between each point (Fig. 8b and c). The results revealed a significant decrease in the HI titer over time at week 1 to 4 and 10 for the herd specific subtype H1C.2.4N2G, which no longer circulated in the herd (mean titer of 731 in week 1 to 132 in week 4 and 48 in week 10 (*p* < 0.001)). However, for the H1N1pdm09 strain actively circulating in the herd, no significant decrease or increase was observed between the samplings.

#### ELISA antibody titers in natural fostered and cross-fostered pigs

**Fig. 9:**
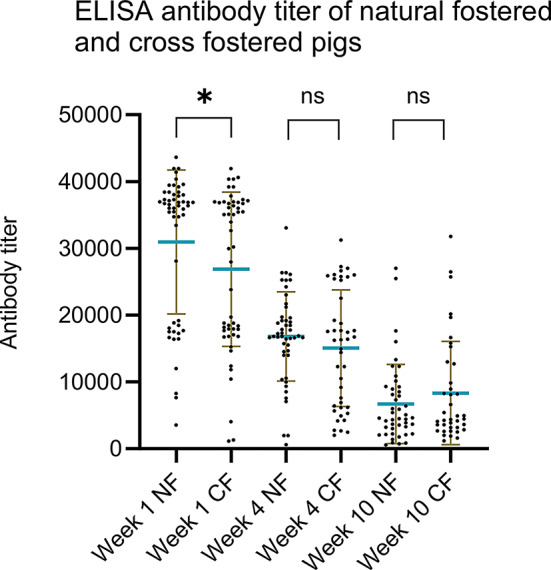
Comparison of the antibody titers of the natural fostered (NF) or cross fostered (CF) pigs. Each point represents a titer of an individual pig. The brown lines indicate error bars and the blue horizontal line indicates the mean titer. The p-value between CF and NF in week 1, 4 and 10, were *p* = 0.0447, *p* = 0.3269 and *p* = 0.5277, respectively

Similar to the longitudinal study a difference in ELISA antibody level between the cross-fostered and naturally fostered piglets were investigated at week 1.The results again showed a significant difference between cross-fostered and naturally fostered pigs in week 1, with the naturally fostered piglets having a mean titer of 30,932 vs. 26,868 in the cross fostered piglets (*p* = 0.045) (Fig. 9).

#### The antibody titers of the sows compared to the piglets

**Fig. 10:**
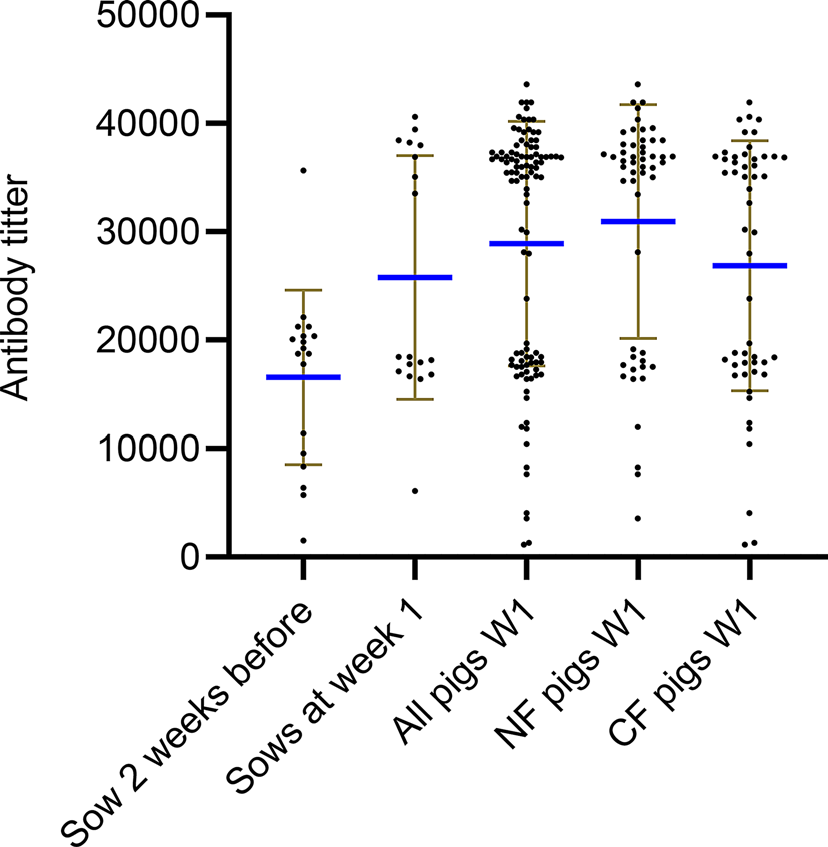
Comparison between the sows ELISA antibody titer two weeks before farrowing and the pigs(all, natural fostered(NF), cross fostered(CF)) ELISA antibody titre. Each point represents a titer of an individual pig. The brown lines indicate error bars and the blue horizontal line indicates the mean titer

To evaluate the relationship between maternal antibody levels and piglet antibody uptake, the week 1 antibody titers of all pigs, as well as naturally fostered and cross-fostered pigs, were compared with the antibody titers of their respective sows (two weeks before farrowing). The mean ELISA antibody titers and the individual ELISA antibody titers are shown in Fig. 10.

To investigate the impact of sow antibody titers on piglet antibody levels at week 1, a Spearman correlation analysis was performed using GraphPad Prism. Piglets were categorised into three groups: naturally fostered, cross-fostered and a combined group. A moderate correlation (*r* = 0.44, *p* < 0.001) was observed in the combined group, stronger than before intervention, suggesting that the intervention enhanced MDA transfer. In addition, different from before the vaccination, in both the natural fostered group and cross-fostered group, a significant correlation (*r* = 0.36, *p* < 0.009 and *r* = 0.53, *p* < 0.001) was observed.

#### Intervention enhanced correlation between ELISA and HI titers for H1C.2.4N2G but not H1N1pdm09

To evaluate the correlation between swIAV ELISA and HI test a Spearman’s correlation analysis was performed. HI titers for the H1C.2.4N2G subtype and the corresponding ELISA antibody titer were included in the analysis, as well as the HI titers for the H1N1pdm09 subtype with the corresponding ELISA antibody titer.

The Spearman correlation made in GraphPad Prism shows a moderate to strong correlation (*r* = 0.565, *p* < 0.001) between the HI titers of the H1C.2.4N2G strain, whereas no significant correlation was observed to the HI titers to the H1N1pdm09 strain.

#### Immunological impact of pre-farrowing vaccination in Herd 2

**Fig. 11:**
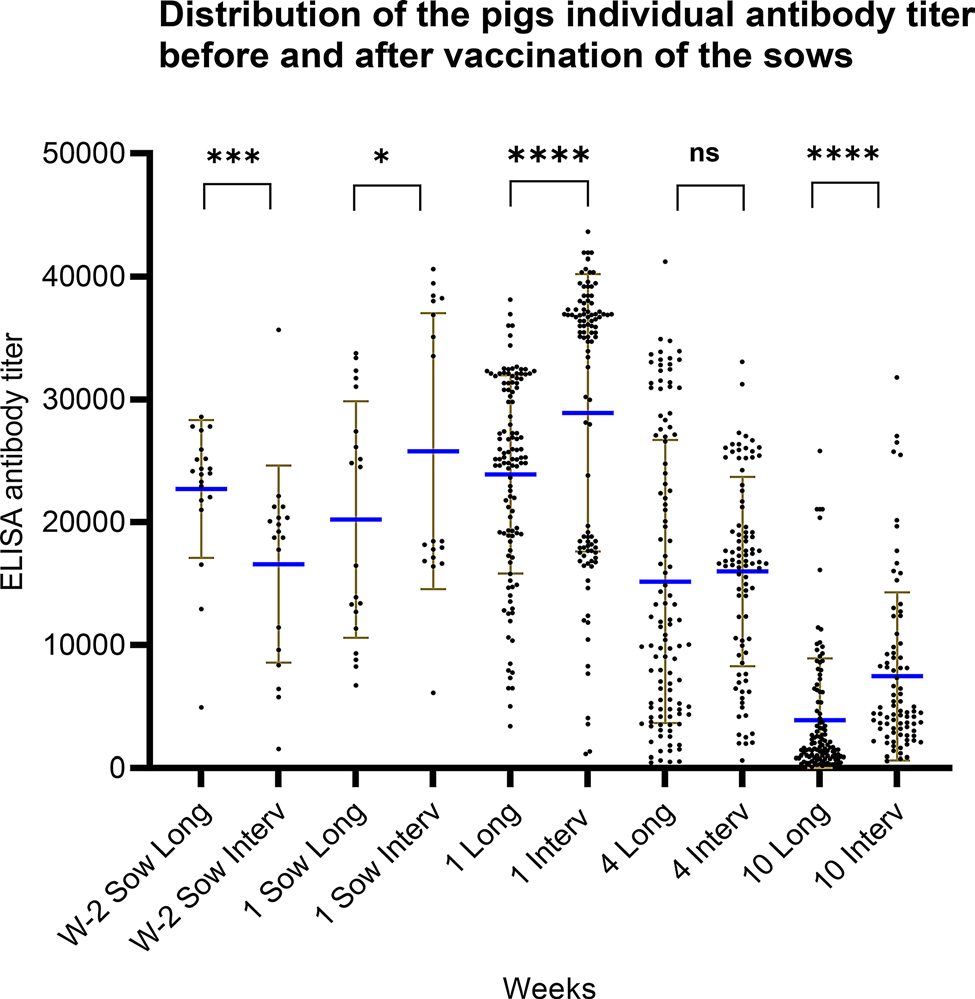
Comparison of the antibody titers of the sows and pigs in the first study (Long) and the intervention study (Interv) in herd 2. Each point represents a titer of an individual pig. The brown lines indicate error bars and the blue horizontal line indicates the mean titer. There is a significant decline between the sows titer two weeks before farrowing (w-2) between the Long and the Interv study(*p* = 0.0004). At week 1 there is a significant increase in the ELISA antibodies of the sows in the intervention study compared to the long study(*p* = 0.0467). For the pigs there are a significant increase in the antibody titers both in week 1(p = < 0.0001) and 10(p = < 0.0001) in the pigs in the intervention study compared to the longitudinal study, and no significant difference was observed in week 4(*p* = 0.2221)

To investigate the antibody titers of the sows and pigs before and after intervention, ELISA antibody titers were compared for the longitudinal and intervention studies, although the same individual sows were not included in both studies. Two weeks before farrowing, the sows in the intervention study had a lower mean ELISA antibody titer of 16281 (Fig. 11, *p* < 0.05) compared to the mean titers of the sows of the longitudinal study that had a mean titer of 22691. In week 1 the sows in the intervention study had a higher ELISA antibody titer with a mean of 25777 than during the longitudinal study with a mean of 20210 *p* < 0.05).

Correspondingly, the piglets of the intervention study had significant higher ELISA titer than the piglets of the longitudinal study at week 1 (mean of 28900 vs. 23893 *p* < 0.05) (Fig. 11). A similar comparison was made based on the HI titers. No significant increase in HI-titers was observed in the sows of the intervention study compared to those of the longitudinal study. However, the piglets had a significant higher HI-titers at week 1 in the longitudinal study against the H1N1pdm09 strain (mean titer 464 in the longitudinal vs. 273 in the intervention study *p* < 0.005), but not against the H1N2 (H1C.2.4N2G) strain (Additional file 5).

## Discussion

This study investigated the infection dynamics of swIAV in two Danish sow herds. In Herd 1, swIAV was detected in only two-thirds of the pigs and was confined to the farrowing unit. In contrast, in Herd 2, nearly all pigs were infected during the study, and two distinct swIAV subtypes and lineages were circulating. H1C.2.4N2G, predominantly circulated in the farrowing unit, and H1N1pdm09, predominantly circulating in the nursery unit, but mixed infection was also observed in a high number of piglets. The management of the piglets in Herd 2 differed from Herd 1 and included several factors that could increase swIAV transmission. For example, Herd 2 had a continuously flow of sows into the farrowing unit, and early weaned piglets stayed in the farrowing unit. Additionally, piglets were not weaned litter-wise but according to size and the location of some stables in the farrowing units required the personal to go through stables with sick pigs and a “baby unit” before entering the other stables. Other factors that was not registered in this study could also play a role in explaining the within-herd differences observed, such as hygiene when handling piglets, level of ventilation, humidity, the flow of employees, introduction of gilts, equipment moved between Sects [33, 57–62]. However, different swIAV strains may behave differently in different herds, depending on the level of herd immunity, the potential match to the vaccine used and the replication efficiency of the virus [38].

The dynamics of swIAV observed in this study were generally consistent with findings from other studies in Danish swine herds [26, 38], showing sustained viral transmission in the farrowing unit and subsequent spillover to the nursery unit. We observed two to three consecutive weeks of viral shedding with the same subtype in the individual piglets, aligning with previous studies that indicate pigs can shed the virus for more than one week [14, 26, 63]. Additionally, a recent review reported an increase in the basic reproduction number (R_0_) for swIAV, supported by an increase in studies reporting prolonged swIAV shedding [64]. Prolonged shedding could be due to suboptimal MDA levels in the individual pigs [14, 65]. While MDAs reduce the susceptibility of piglets to swIAV infection, they do not necessarily affect swIAV shedding, and shedding duration may be longer or similar to that observed in seronegative pigs [24, 60, 65].

Other studies with extended sampling periods, such as sampling twice in the farrowing unit and more frequently in older age groups or sampling the herds every 30 days, have provided deeper insights into infection dynamics over time [2, 11, 18, 23, 26, 63, 66, 67]. These studies typically found that only one subtype circulates within herds [12], though some have reported the simultaneous circulation of two strains [6, 19, 68]. This aligns with our findings, where we observed the introduction of H1N1pdm09 in the middle of the first study in Herd 2. Similarly, the Danish passive surveillance has reported that 7% of herds have more than one subtype circulating at the same time [69]. However, we did not detect the initial H1C.2.4N2G subtype circulating during the intervention study, which together with the fact that most herd in the Danish swIAV surveillance only detect a single strain, could suggest that over time one swIAV strain will dominate.

A high proportion of the piglets were infected with swIAV despite nearly all pigs testing positive for swIAV antibodies by the ELISA in quite high levels at week 1. The lack of effect on swIAV infection of MDAs detected by ELISA has also been shown in previous studies [2, 14, 24, 26]. Moreover, Deblanc et al. [24] reported that MDAs delayed the immune response and did not reduce swIAV shedding. However, another study [70] reported that piglets with MDA had milder clinical signs than pigs without MDA during their first infection, but also suggested that MDA inhibited an active immune response after swIAV infection. In this study, the lack of increase in swIAV antibody titer after infection was also observed. In Herd 1, both ELISA and HI antibodies titers decreased over time independent of the early infection with swIAV in the farrowing unit, at a time where the MDA level was high. This correlates with previous studies showing that a high level of MDA can limit the active immune response as they inhibit early-life antibody responses [71]. This also aligns with our results, showing both nasal discharge, cough and sneezing in swIAV infected piglets despite the presence of MDAs.

In Herd 2, two swIAV strains circulated in both the farrowing- and nursery unit, with the H1N1pdm09 strain infecting pigs at a later time point. The ELISA antibody titers exhibited a trend similar to that observed in Herd 1, characterized by high levels early in life followed by a subsequent decline. In contrast, HI antibody titers remained stable against the H1C.2.4N2G strain, while a slight increase was noted against H1N1pdm09. This different HI antibody pattern between Herd 1 and 2, could reflect that the piglets were exposed to swIAV later in Herd 2, where the MDAs were already declining, which could impact the number of pigs experiencing some level of active immunity to the herd strains. This was specifically evident for the H1N1pdm09, which was also causing the later infections, and here an increase in HI-titers were observed after infection as expected if the level of MDAs is low. In general, the lack of seroconversion following swIAV infections is problematic for the endemic circulation of swIAV, as it may leave pigs susceptible to reinfections with the same swIAV strain. Some studies have provided evidence of reinfection with the same swIAV strain [14, 72].

This study did not find a difference in the occurrence of swIAV infection between cross-fostered and naturally fostered piglets, which might be explained by the fact that litter equalization was performed within the first 24 h, where swIAV is not yet detected in the piglets. Nevertheless, our results suggests that naturally fostered piglets had a greater uptake of swIAV antibodies (from colostrum) compared to cross-fostered piglets, which could be a result of disrupted colostrum intake in cross-fostered piglets. The disruption could be attributed to the piglets being moved at a very crucial time-point for intestinal antibody uptake through the colostrum. It is known that especially, the first six hours are very important for the uptake [25]. Additionally, they might spend considerable time outside the pens, waiting to be allocated to a new sow and when arriving they need to establish a new hierarchy. A decreased colostrum intake decreases the change of survival [73] and potentially make them more susceptible to infectious diseases including swIAV. Interestingly, another study found a significant increased risk of swIAV infections in cross-fostered litters [58]. However, in that study, whole batches with and without cross-fostering was compared, which might make it more likely to observe a difference in the litter compared to our study. Furthermore, a field study examining the effects of cross-fostering on performance, clinical health, and antibiotic usage [74] revealed that strategies that limit cross-fostering could enhance piglet health and reduce antibiotic usage further emphasizing that the consequences of cross-fostering vs. the benefits should be evaluated. In the intervention study, higher swIAV antibodies titers were observed in the piglets of both cross-fostered and naturally fostered piglets, indicating that pre-farrowing vaccines could decrease the impact of a lower colostrum intake if the average titers in sows are higher. However, other factors such as management in the farrowing unit, sow parity and the robustness of the piglets could have influenced the results.

For the intervention study, the pre-farrowing vaccination did seem to increase the level of swIAV ELISA antibody titers in the vaccinated sows when comparing the samples obtained two weeks before farrowing and one week after farrowing. However, the HI-titer was not significantly increased in the sows. This could indicate that the inactivated vaccine mainly stimulates specific types of antibodies including NP antibodies, whereas the neutralising HI antibodies were not increased. This could explain why, despite the high ELISA titers, piglets were still infected with H1N1pdm09 even at one week of age after the pre-farrowing vaccinations. Additionally, an unwarranted effect of high MDAs level was noted as a higher number of piglets showed consecutive shedding and no increase in swIAV antibodies following infection after the pre-farrowing vaccination was applied. The fact that MDAs are not able to hinder swIAV infection in the upper respiratory tract is not surprising. It is only IgG from the colostrum that can cross the intestinal barrier, and IgG circulate systemically, and can therefore provide a potential protection of the lungs. However, limited mucosal immunity is stimulated, and several studies have shown that mucosal IgA is needed to hinder swIAV infection of the nasal cavity [75–77]. In addition to the limited effect that MDAs have on virus shedding, several herd characteristics also play a major role in the ability to observed potential effects of vaccinations. In Herd 2, several factors were present that could enhance the virus transmission, and despite the efforts applied in the intervention study in management procedures, the daily flow of workers through a sick unit and baby unit to access the farrowing stables probably overturns the potential beneficial effects of hand disinfectants and litter-wise weaning etc. Additionally, the homology between herd and vaccine strain can also play a crucial role on the effect of vaccination [30], and in our study major differences were observed to the vaccine strains.

In the longitudinal study, a correlation between swIAV shedding and nasal discharge was found, whereas in the intervention study no significant difference was observed. This result may suggest that sow vaccination reduces clinical signs in pigs, as the RespiporcFlu3 vaccine [27] claims to provide protection against clinical signs for 33 days with MDA, although it does not prevent infection. The lack of statistical significance in the intervention study may be attributed to the sample size. A larger sample size could yield different results. However, it is also plausible that sow vaccination contributed to the reduction in nasal discharge. Further research, ideally including a case-control group in field settings, is necessary to validate these findings. In general, it should also be noted that this study only included two Danish sow herds and as the results illustrated swIAV behaved differently in both herds, which makes it challenging to make generalizing statements on swIAV dynamics.

Regarding the impact of swIAV on weight gain, no significant difference was found in our study. This could be attributed to the study design, particularly the absence of a final weight measurement before the pigs were moved out of the nursery unit. Notably, many pigs in Herd 2 were infected with swIAV after their last weight measurement in week 4 or at the same time. A previous study reported a weight difference of approximately 1 kg between swIAV-infected and non-infected pigs at week 6 [14], and several other studies similarly have demonstrated that swIAV can affect feed intake and weight gain [13, 78], emphasising that control of swIAV is essential not only for improving animal welfare but also for enhancing production efficiency and reducing economic losses.

## Conclusion

This study revealed that swIAV infection dynamics vary greatly among herds. Despite following six different batches, four of which were from the same herd, there was no consistency in the transmission patterns across all batches. The study also documented that antibody levels decreased even though the pigs were infected with swIAV and that cross-fostering can result in lower antibody titers at week 1.

In the intervention study, vaccinating sows two weeks before farrowing, implementing extra hygiene measures, and making management changes did not have a great impact on swIAV circulation, but did result in a significantly higher antibody titer in one-week-old piglets. However, this higher antibody titer potentially increased the number of prolonged swIAV shedders and limited the number of pigs seroconverting after swIAV infection further sustaining the endemic status of swIAV.

## Supplementary Information

Below is the link to the electronic supplementary material.

Supplementary Material 1

Supplementary Material 2

Supplementary Material 3

Supplementary Material 4

Supplementary Material 5

## Data Availability

All relevant data are included in the manuscript and its supplementary files.
