# Supplementary material for: Influenza A virus infection dynamics in two sow herds and effects of interventions
Source: Porcine Health Manag. 2026 Jan 13;12:19. doi: 10.1186/s40813-025-00481-2 (PMC13081247; doi:10.1186/s40813-025-00481-2)
Supplement: Supplementary file 1 — Supplementary Material 1 [file 40813_2025_481_MOESM1_ESM.docx]

**Description of Reverse transcription and pre-amplification**

| RNA |
| --- |
| A total of 7.5 µL of 2x RT‒PCR buffer was mixed with 1.0 µL of 200 nM primer mixture (containing all the different primer sets listed in additional file 2), 0.6 µL of 25x RT‒PCR mixture, 2.90 µL of RNase-free water and 3 µL of RNA. The combined one-tube with reverse transcription and pre-amplification was performed on a PCRmaxTM Alpha Cycler 2 PCR Machine (Alpha Thermal Cycler, Cole-Parmer Ltd., UK) with the following conditions: 20 minutes at 45°C, 10 minutes at 95°C, 24 cycles of 94°C for 15 seconds and 60°C for 45 seconds. The pre-amplified complementary DNA (cDNA) was stored at -20°C. |
| DNA |
| The reaction contained 5 µL of TaqMan PreAmp Master Mix, 2.5 µL of 200 nM primer mixture (containing all the different primer sets listed in additional file 1) and 2.5 µL of DNA. Pre-amplification was performed on a PCRmaxTM Alpha Cycler (Alpha Thermal Cycler) with the following thermal cycling conditions: 95°C for 10 minutes, 14 cycles of 95°C for 15 seconds and 60°C for 4 minutes. The pre-amplificated DNA was stored at -20°C. |
