# Supplementary material for: Influenza A virus infection dynamics in two sow herds and effects of interventions
Source: Porcine Health Manag. 2026 Jan 13;12:19. doi: 10.1186/s40813-025-00481-2 (PMC13081247; doi:10.1186/s40813-025-00481-2)
Supplement: Supplementary file 3 — Supplementary Material 3 [file 40813_2025_481_MOESM3_ESM.docx]

**Comparison of the HI titers of the natural fostered(NF) or cross fostered(CF) pigs in both Herd 1 and Herd 2.** The HI titers are shown for the herd specific subtypes that was found in the herds. Each point represents a titer of an individual pig. The brown lines indicate error bars and the blue horizontal line indicates the mean titer.
