# Supplementary material for: Influenza A virus infection dynamics in two sow herds and effects of interventions
Source: Porcine Health Manag. 2026 Jan 13;12:19. doi: 10.1186/s40813-025-00481-2 (PMC13081247; doi:10.1186/s40813-025-00481-2)
Supplement: Supplementary file 4 — Supplementary Material 4 [file 40813_2025_481_MOESM4_ESM.docx]

**HI titer for both subtypes found in Herd 2 before and after intervention.** Long indicates the first study in Herd 2 and Interv indicates the intervention study. Each point represents the titer of an individual pig. The brown lines indicate error bars, and the blue horizontal line indicates the mean titer.
