# Supplementary material for: Influenza A virus infection dynamics in two sow herds and effects of interventions
Source: Porcine Health Manag. 2026 Jan 13;12:19. doi: 10.1186/s40813-025-00481-2 (PMC13081247; doi:10.1186/s40813-025-00481-2)
Supplement: Supplementary file 5 — Supplementary Material 5 [file 40813_2025_481_MOESM5_ESM.docx]

Table 1: **List of primers and probes used in the high-throughput RT-PCR for swIAV testing and subtyping.** Each target gene used is represented in the first row, hereafter the primer/probe names, then the sequences for each primer/probe, the length and as the last part a reference of where the sequences are used from or modified from.

| **Virus/target gene** | **Primer Name/probe** | **Sequences** | **Length(pb)** | **References** |
| --- | --- | --- | --- | --- |
| **Swine influenza A virus**  **SIV (M)** | M-F  M-R  M-P | **AGATGAGT**CTTCTAACCGAGGTCG  TGCAAAGACACTTTCCAGTCTCTG  FAM-TCAGGCCCCCTCAAAGCCGA-**BHQ1** | 101 | (45) |
| **H1pdm** | H1pdm | CTA GTG GTA CCG AGA TAT GCA  TAT TGC AAT CGT GGA CTG GTG T  [FAM] CGC AAT GGA AAG AAA TGC TGG ATC TGG [BHQ1] |  | (6) |
| **H1pdm** | NIID-swH1-F1DK  NIID-swH1-R1DK  NIID-swH1-P2dk | AGA AAA RAA TGT AAC AGT AAC ACA CTC YGT  TGY TTC CAC AAT GTA GGA CCA T  [FAM] CAK CCA GCA ATR TTA CAT TTA CC [MGBEQ] |  | (48) |
| **H1N1pdm09** | H1fw2sw-2  H1rev2sw-2  H1probe2sw-2 | AGT TCA AGC CGG AAA TAG CA  CCC GGC TCT ACT AGT GTC CA  [FAM] CCC AAA GTG AGG RAT CAA GAA GGG AG [BHQ1] | 87 | (44) |
| **H1av** | H1av-F  H1av-R  H1av-P | GAAGGRGGATGGACAGGAATGA  CAATTAHTGARTTCACTTTGTTGCTG  FAM-TCTGGTTACGCAGCWGATCAGAAAA-BHQ1 | 139 | (44) |
| **H3hu** | H3hu-F  H3hu-R  H3hu-P | TGATGGAGAAAACTGCACACTA  CGTTCAACAAAAAGGTCCCATTTC  FAM-CACACTGAGGGTCTCCCAATAGAGCATCTA-BHQ1 | 93 | (44) |
| **H3sw** | H3sw-F  H3sw-R  H3sw-P | TGATGGAGCAAATTGCACACTG  CGTTCAATGAAAAGGTCCCATTTC  FAM-CACAATGAGGGTCCCCTAATAGAGCGTCCA-BHQ1 | 93 | (44) |
| **N1pdm** | N1pdm-F  N1pdm-R  N1pdm-P* | CGAAATGAGTGCCCCTAATTATC  CGATTCGAGCCATGCCAGTTA  FAM-[+C][+C]T[+G]ATTCT[+A]GTGAAATCA[+C]-BHQ1 | 102 | (44) |
| **N1** | N1-F  N1-R | CCTTGCTTCTGGGTTGAACTAATC  AGTGTCACTATTTACACCACAAAAGG | 99 | (44) |
| **N2sw** | N2-F  N2-R | GAGTATGGTGGACBTCAAAYAG  TTGCGAAAGCTTATATAGGCATGA | 101 | (6) |
| **N2hu** | N2hu-F  N2hu-R | CTGGTATTTTCTCTGTTGAAGGC  CCASACTTCAKTTTCCTGYTTCC | 92 | (44) |
